# Integrated RNA-seq and DNase-seq analyses identify phenotype-specific BMP4 signaling in breast cancer

**DOI:** 10.1186/s12864-016-3428-1

**Published:** 2017-01-11

**Authors:** M. Ampuja, T. Rantapero, A. Rodriguez-Martinez, M. Palmroth, E. L. Alarmo, M. Nykter, A. Kallioniemi

**Affiliations:** 1 BioMediTech, University of Tampere, Tampere, Finland; 2 Fimlab Laboratories, Tampere, Finland

**Keywords:** Bone morphogenetic protein, Breast cancer, NGS, RNA-seq, DNase-seq, Transcription factor

## Abstract

**Background:**

Bone morphogenetic protein 4 (BMP4) plays an important role in cancer pathogenesis. In breast cancer, it reduces proliferation and increases migration in a cell line-dependent manner. To characterize the transcriptional mediators of these phenotypes, we performed RNA-seq and DNase-seq analyses after BMP4 treatment in MDA-MB-231 and T-47D breast cancer cells that respond to BMP4 with enhanced migration and decreased cell growth, respectively.

**Results:**

The RNA-seq data revealed gene expression changes that were consistent with the in vitro phenotypes of the cell lines, particularly in MDA-MB-231, where migration-related processes were enriched. These results were confirmed when enrichment of BMP4-induced open chromatin regions was analyzed. Interestingly, the chromatin in transcription start sites of differentially expressed genes was already open in unstimulated cells, thus enabling rapid recruitment of transcription factors to the promoters as a response to stimulation. Further analysis and functional validation identified MBD2, CBFB, and HIF1A as downstream regulators of BMP4 signaling. Silencing of these transcription factors revealed that MBD2 was a consistent activator of target genes in both cell lines, CBFB an activator in cells with reduced proliferation phenotype, and HIF1A a repressor in cells with induced migration phenotype.

**Conclusions:**

Integrating RNA-seq and DNase-seq data showed that the phenotypic responses to BMP4 in breast cancer cell lines are reflected in transcriptomic and chromatin levels. We identified and experimentally validated downstream regulators of BMP4 signaling that relate to the different in vitro phenotypes and thus demonstrate that the downstream BMP4 response is regulated in a cell type-specific manner.

**Electronic supplementary material:**

The online version of this article (doi:10.1186/s12864-016-3428-1) contains supplementary material, which is available to authorized users.

## Background

Despite many advances in diagnostics and therapeutics, breast cancer remains the leading cause of cancer death in women [1]. Bone morphogenetic proteins (BMPs) are a group of growth factors that are important players during development [2, 3] but also contribute to cancer formation and progression [4–6]. As a subfamily of the transforming growth factor β (TGF-β) protein superfamily, BMPs are extracellular ligands that bind as dimers to their specific transmembrane receptors and activate the intracellular SMAD signaling pathway leading to phosphorylation of receptor-regulated SMADs (SMAD1/5/9). The activated SMADs bind to SMAD4 and the complex translocates to the nucleus where it regulates the expression of BMP target genes [7, 8]. Alternatively, BMP signals are also mediated through the activation of ERK, JNK and p38 mitogen-activated protein kinase pathways [7, 8].

The functional consequences of BMP signaling depend on the BMP ligand and tissue type. We and others have shown that BMP4 reduces the proliferation of breast cancer cell lines, while simultaneously inducing migration and invasion in a subset of cell lines [9–11]. Similar dualistic effects upon BMP4 stimulation have also been reported in other tumor types [12]. Concordantly, data from breast cancer patient samples point to a correlation between elevated BMP4 levels and reduced proliferation as well as an increased risk of recurrence [13]. These BMP4-related effects that seem either detrimental (reduced cell growth) or beneficial (increased mobility) for the cancer cells are likely to be mediated by specific BMP4 target genes. The identification of such target genes is thus important since it may allow generation of effective cancer therapies targeting each phenotype independently.

We have previously searched for BMP4 target genes in a set of breast cancer cell lines that predominantly respond to BMP4 treatment by reduction of proliferation [14]. Here, we used next-generation sequencing (NGS) technologies (RNA-seq and DNase-seq) to uncover BMP4-mediated transcriptional events with a specific focus on comparing cells in which BMP4 has opposing effects, namely antiproliferative and promigratory. Out of the nine breast cancer cell lines we have previously studied, T-47D shows one of the most prominent growth reductions and MDA-MB-231 cells display the most overt induction of migration [9, 10], and were thus selected for this study.

RNA-seq method quantifies the level of gene expression across the genome [15] while DNase-seq allows identification of open chromatin regions that are sensitive to digestion by the DNase I endonuclease [16]. Open chromatin regions are considered as sites where transcriptional regulation can take place since they are accessible for regulatory molecules to bind and exert their function. By combining data from RNA-seq and DNase-seq, and using additional data analysis tools, it was possible to identify candidate transcription factors involved in the observed transcriptional responses. This approach thus provides the means to better understand the transcriptional events that link BMP4 signaling and its resulting phenotypes.

## Results

We performed RNA-seq and DNase-seq analyses in two breast cancer cell lines, T-47D and MDA-MB-231. The cell lines were treated with BMP4 and vehicle control for 3 h, thus allowing us to specifically focus on early response events. Both vehicle- and BMP4-treated cell lines were sequenced (see methods).

### BMP4-elicited transcriptional regulation is highly divergent in the two breast cancer cell lines with different functional responses to BMP4

Sequencing reads from RNA-seq and DNase-seq were aligned to the human genome and further analyzed as described in the methods. To confirm that the two datasets were consistent, we compared the chromatin openness as determined by DNase-seq signal at the transcription start site (TSS) to the expression level of the gene as determined by RNA-seq. As expected, we found that the increased openness of TSS globally correlated with increased gene expression (Additional file 1: Figure S1, Panels A and B). However, the variance is high, indicating that the differences in the chromatin state only partly explain gene expression patterns.

**Fig. 1:**
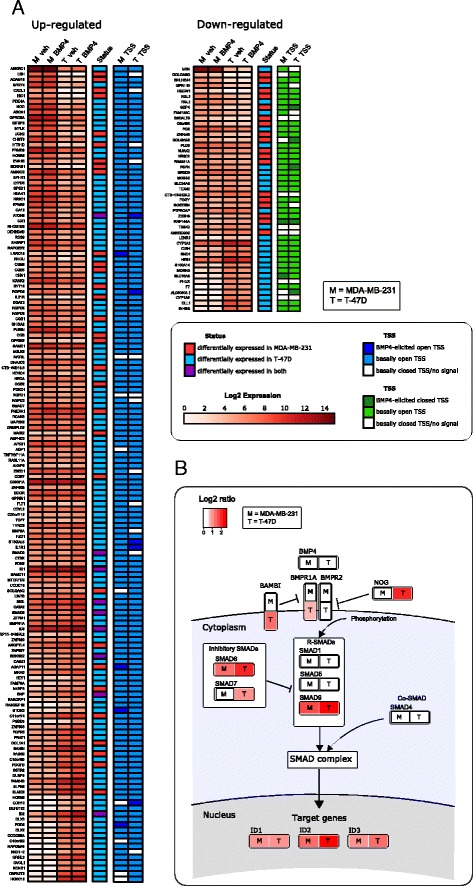
The RNA-seq and DNAse-seq data reveal cell line-specific responses to BMP4. **a** Gene expression levels of differentially expressed protein-coding genes converted to log2 scale are shown for both cell lines and treatments, upregulated genes on the *left* and downregulated genes on the *right*. The status column denotes the cell line in which the gene is differentially expressed. The rightmost columns indicate the status of the chromatin at transcription start sites (TSS) of the DEGs as measured by DNAse-seq. **b** Illustration of the differentially expressed components of the BMP signaling pathway upon BMP4 treatment

Next we compared the expression levels from RNA-seq between the vehicle- and BMP4-treated cells. This analysis identified 91 differentially expressed genes (DEGs) in MDA-MB-231, of which 58 were upregulated and 33 downregulated (Additional file 2: Table S1). In T-47D, there were 203 DEGs, of which 160 were upregulated and 43 were downregulated (Additional file 3: Table S2). In total, 10 DEGs (*ATOH8*, *BDKRB2*, *BMF*, *GS1-124 K5.4*, *ID1*, *ID2*, *ID3*, *SKIL*, *SMAD6*, and *SMAD9*) were shared by the two cell lines and all of them were upregulated except *GS1-124 K5.4* which was downregulated in both cell lines. To illustrate that BMP4 induces markedly divergent transcriptional responses in these two cell lines, we generated a heatmap to show the expression levels of the protein-coding DEGs (Fig. 1a). Using the DNase-seq data, we examined the chromatin status at the transcription start sites (TSSs) of these protein-coding DEGs. For the majority of the cases the chromatin was open at the TSS before BMP4 stimulation (approximately 86% of all DEGs in both cell lines) (Additional file 1: Figure S1, Panels C and D). For the remaining DEGs, we observed either opening or closing of the TSS after stimulation or no change in the closed chromatin status (Fig. 1a). These data indicate that, at this early time point, the BMP4-induced differential expression mainly involves genes whose transcription does not require changes in the chromatin status at TSS.

The DEG lists included a number of genes involved in the canonical BMP pathway. As expected, *ID1, ID2* and *ID3*, known BMP4 target genes, were upregulated in both cell lines (Fig. 1b). Similarly, the receptor-regulated *SMAD9* was upregulated in both cell lines whereas no significant difference in the other receptor-regulated SMADs or *SMAD4* expression was observed. Among the inhibitory SMADs, *SMAD*6 was upregulated in both cell lines and *SMAD7* in T-47D. In addition, the BMP type I receptor *BMPR1A* and negative regulators of BMP signaling, *NOG* and *BAMBI*, were upregulated in T-47D while in MDA-MB-231 their expression was not significantly changed (Fig. 1b). Thus BMP4 stimulation leads to expression changes having characteristics of both feedback and feedforward loops.

We then evaluated whether the differentially expressed genes participate in specific biological processes and especially assessed whether the non-common DEGs have differing functions. To this end we used DAVID to search for GO terms enriched in the sets of non-common protein-coding DEGs. In MDA-MB-231, most of the enriched terms were related to cell migration whereas organ development and morphogenesis as well as intracellular signaling were the most significant GO terms in T-47D (Table 1). These findings imply that the transcriptional changes are indeed likely to explain the dissimilarities in the phenotypic responses of these two cell lines to BMP4 treatment.

**Table 1:** Gene ontology analysis

*Cell line*	*GO accession*	*GO term*	*Number of genes*	*Adjusted p-value*
*MDA-MB-231*	GO:0030334	regulation of cell migration	5	2.0 × 10^−2^
	GO:0030335	positive regulation of cell migration	4	2.3 × 10^−2^
	GO:2000145	regulation of cell motility	5	2.4 × 10^−2^
	GO:2000147	positive regulation of cell motility	4	2.5 × 10^−2^
	GO:0051272	positive regulation of cellular component movement	4	2.7 × 10^−2^
*T-47D*	GO:0048513	animal organ development	45	2.6 × 10^−8^
	GO:0035556	intracellular signal transduction	41	4.5 × 10^−8^
	GO:0009887	organ morphogenesis	22	4.0 × 10^−7^
	GO:0009966	regulation of signal transduction	36	4.5 × 10^−6^
	GO:0007166	cell surface receptor signaling pathway	34	9.5 × 10^−5^

The DAVID Functional Annotation Tools was used to reveal significantly enriched GO categories among the differentially expressed protein-coding genes. The analysis was done independently for each cell line and shared differentially expressed genes were omitted. The top five biological function GO terms are shown

Thereafter, we also wanted to investigate whether the expression levels of DEGs could be linked with survival in breast cancer patients. For this purpose, we used the data publicly available in the TCGA database. The results showed that 20 DEGs in the MDA-MB-231 and 46 DEGs in the T-47D cells associated with either good or poor prognosis (Additional file 4: Tables S3 and S4). Of the nine shared protein-coding DEGs, four (*ATOH8*, *ID3*, *SMAD6* and *SMAD9*) were correlated with survival, all being associated with poor prognosis.

**Fig. 2:**
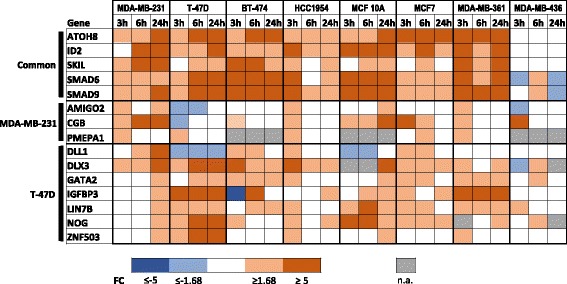
Expression levels of selected BMP4 target genes by qRT-PCR in a breast cancer cell line panel. The expression levels of 15 DEGs were measured after 3, 6 and 24 h of BMP4 treatment in the indicated cell lines. The color code illustrates the relative expression levels in the BMP4-treated sample as compared to the corresponding vehicle control. FC = Fold change, n.a. = mRNA level too low to allow reliable measurement

To validate the results of the RNA-seq analysis and to extend the scope of the study beyond the 3 h time point in two cell lines, qRT-PCR was used to study the expression levels of 15 selected DEGs in MDA-MB-231 and T-47D cells as well as in five additional breast cancer cell lines (BT-474, HCC-1954, MCF-7, MDA-MB-361, and MDA-MB-436) and one normal breast epithelial cell line (MCF-10A) treated with BMP4 and vehicle for 3, 6 and 24 h. The genes were selected based on their expression levels and reported cancer association in the literature, and five of these were upregulated according to the RNA-seq in both MDA-MB-231 and T-47D. The expression patterns of the majority of the genes showed similarities across the cell line panel and time points with the clear exception of MDA-MB-436, in which the expression changes were very limited (Fig. 2). Particularly the five shared genes (*ATOH8*, *ID2*, *SKIL*, *SMAD6* and *SMAD9*) as well as *DLX3* were consistently upregulated upon BMP4 treatment throughout the time series thus confirming that they represent common BMP4 target genes. The remaining genes showed more variability with altered expression typically in only two to three cell lines, suggesting that their expression is likely to be influenced by factors that are cell line-specific.

### Chromatin landscape and dynamics following BMP4 treatment

To gain more insight into the changes of chromatin structure during BMP4 treatment, we performed peak detection in a genome-wide manner to identify the areas of open chromatin. The peak detection approach was benchmarked by comparison to publicly available DNase-seq data of unstimulated T-47D cell line from ENCODE (see methods), showing that most of the peaks identified in our data are present also in ENCODE samples (Additional file 5: Table S5).

After filtering procedures (see methods), the numbers of identified DNase hypersensitive sites (DHSs) in the MDA-MB-231 cell line were 89,830 and 97,349 in vehicle- and BMP4-treated samples, respectively. In T-47D, the corresponding numbers were 68,000 and 73,881. To obtain a unified set of peaks for both conditions, the overlapping DHSs were merged resulting in a total of 106,154 DHSs in MDA-MB-231 and 110,028 in T-47D. After the merging, the fraction of shared DHSs between BMP4 and vehicle control in MDA-MB-231 samples was 75% while the fraction of unique DHSs in the vehicle was 9% and correspondingly in the BMP4 sample 16% (Additional file 6: Figure S2). In the T-47D cell line, the fraction of shared DHSs between the two conditions was 27% whereas the fraction of unique DHSs in the vehicle was 34% and in the BMP4 sample 39% (Additional file 6: Figure S2).

**Fig. 3:**
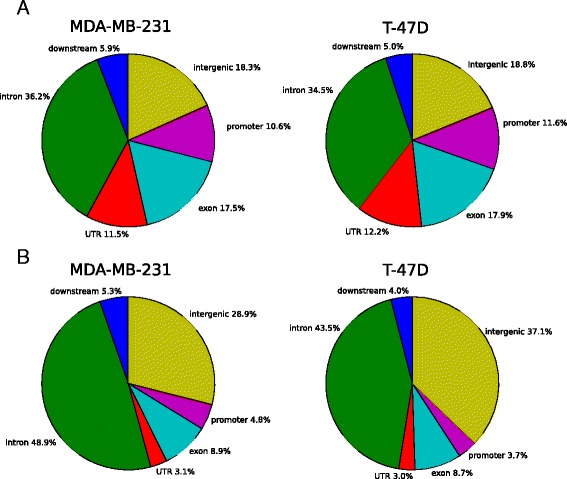
Distribution of open chromatin regions. Annotation of open chromatin regions in MDA-MB-231 and T-47D after (**a**) vehicle treatment (basal openness) and (**b**) BMP4 treatment (consisting only of the chromatin that opened after BMP4 treatment)

Annotation of the merged DHSs to genomic features revealed a similar distribution in the two cell lines in the vehicle-treated condition, with the largest fraction (>30%) of DHSs locating in introns (Fig. 3a). When comparing the distributions of the BMP4-induced DHSs between the cell lines apparent resemblances were also observed. In both cell lines, the proportion of DHSs associated with intronic and intergenic regions increased after BMP4 stimulation with a corresponding decrease at other genomic locations, including the promoter regions (Fig. 3b).

To assess the functional impact of the BMP4-induced global changes in the chromatin structure we conducted an enrichment analysis using GREAT [17] which maps the DHSs to putative regulatory regions of genes and conducts a gene ontology enrichment analysis. The results highlighted e.g. cell motility and organ morphogenesis as enriched biological functions for MDA-MB-231 and T-47D, respectively (Additional file 7: Tables S6 and S7). These results are consistent with those obtained by enrichment analysis of the differentially expressed genes from RNA-seq (Table 1) and thereby suggest that, together with specific target genes, BMP4-induced changes at chromatin level may contribute to the emergence of the different BMP4-mediated phenotypes.

### Transcription factor binding site enrichment analysis in open chromatin regions of promoters reveals transcription factors involved in BMP4 signaling regulation

Based on our TSS openness analysis (Fig. 1a), a dominant feature of our data is that the chromatin of the putative BMP4 target genes (identified by RNA-seq) is open already in vehicle-treated cells. This is further supported by our genome-wide peak analysis, where the promoter regions were not overrepresented after the treatment (Fig. 3b). Therefore, the alterations in the chromatin state only partially explain gene expression differences induced by the BMP4 treatment. However, differential transcription factor binding to open promoters may explain the different responses in the cell lines. Therefore we performed transcription factor (TF) motif binding analysis. To assess which TFs might be regulators of the BMP4 response, the sequences of open chromatin sites in the proximal promoters of upregulated genes were analyzed with a total of 426 position weight matrixes (PWMs), representing 401 individual TFs or TF-complexes (see methods). For each TF we calculated an enrichment score (see methods) for the number of binding sites in either MDA-MB-231 or T-47D cells.

**Fig. 4:**
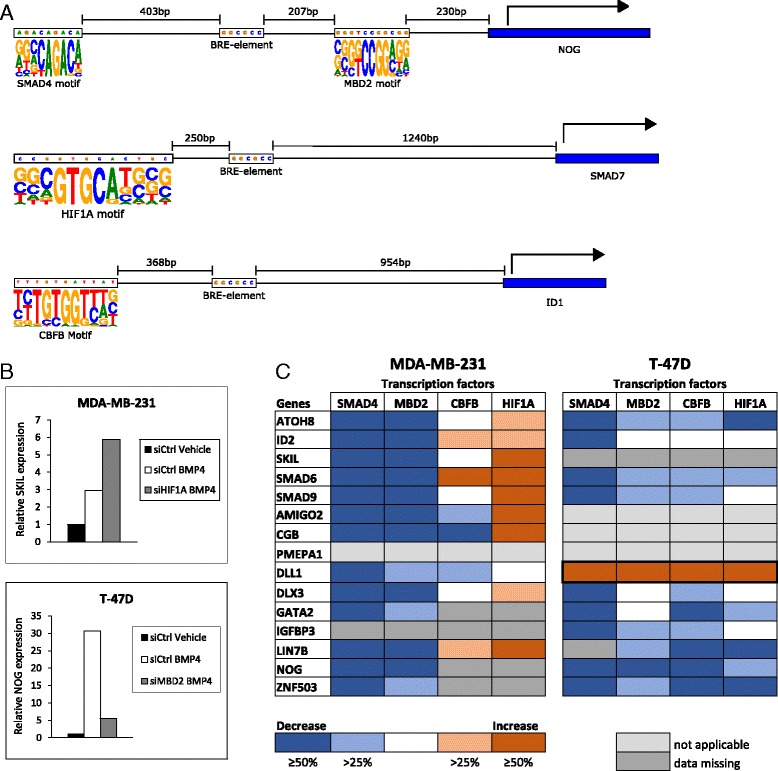
Examples of predicted TFBSs and the impact of transcription factors on BMP4 target gene expression. **a** The predicted binding sites of transcription factors MBD2, HIF1A and CBFB are depicted at the promoters of *NOG*, *SMAD7* and *ID1* genes, respectively. In addition, known BMP-response elements (BRE) located near the binding sites are illustrated. **b** The TFs were silenced and the cells were treated with BMP4 or vehicle control followed by measurement of target gene expression by qRT-PCR. Examples of relative expression levels of *SKIL* after HIF1A silencing in MDA-MB-231 cells (*top panel*) and *NOG* expression after MBD2 silencing in T-47D cells (*bottom panel*) are shown. **c** Graphical summary of the TF silencing experiments. The order of the genes is identical to that in Fig. 2. Blue color (decreased target gene expression) denotes TFs that were essential for target gene expression and red color those whose silencing led to enhanced target gene expression. Not applicable indicates cases where BMP4 did not alter the baseline gene expression. Data on the DLL1 gene, which is downregulated in T-47D upon BMP4 treatment, are highlighted with a *bold line*

This analysis led to the identification of candidate regulator TFs, including multiple members of the SMAD family of TFs, as expected, as well as a number of shared common regulator TFs. To focus on biologically relevant candidates, we filtered out those TFs that were not expressed based on our RNA-seq data. In addition, we included only those TFs whose binding sites (TFBSs) in open chromatin regions of the promoters of DEGs were enriched in one and depleted in the other cell line. The top 15 TFs that are expressed in both cell lines but have a high enrichment score only in one of the cell lines are listed in Tables 2 and 3. Examples of target gene promoters with binding motifs for predicted TFs are shown in Fig. 4a.

**Table 2:** Top 15 transcription factors enriched in MDA-MB-231 cells

*TF name*	*Motif*	*Selection by:*	*TF binding sites*	*Ref. sites*	*Expected sites in ref.*	*Ratio of enrichment*	*Mean read count*
*MYBL2*	MYBB_f1	2, 3, 4	12	2930	6.2	1.92	2197
*BACH1*	BACH1_si	1, 2, 3	15	3904	8.3	1.81	531
*MYC*	MYC_f1	1, 2, 4	10	2698	5.7	1.74	3044
*MAFK*	MAFK_si	2, 3	16	4428	9.4	1.70	688
*RELA*	TF65_f2	1, 2, 4	19	5467	11.6	1.63	1398
*PPARA*	PPARA_f1	1, 2, 3	9	2747	5.8	1.54	185
*NFIA/B/C/X*	^a^	1, 2, 3	15	4669	9.9	1.51	^b^
*NFIL3*	NFIL3_si	1, 2, 3	11	3494	7.4	1.48	474
*FOXA2*	FOXA2_f1	1, 2, 3	36	11477	24.4	1.47	434
*REL*	REL_do	1, 2, 3	17	5422	11.5	1.47	69
*ZFHX3*	ZFHX3_f1	2, 3	46	14683	31.2	1.47	66
*RXRB*	RXRB_f1	1, 2, 4	20	6414	13.6	1.47	1015
*SMARCC1*	SMRC1_f1	1, 4	20	6443	13.7	1.46	1478
*ETV5*	ETV5_f1	2, 3	16	5199	11.1	1.45	641
*NR3C1*	GCR_si	1, 2, 4	15	4910	10.4	1.44	1087

The ratio of enrichment is the result of dividing the number of TF binding sites by the number of expected sites. Motifs are derived from the HOCOMOCO database. ^a^NFIA + NFIB + NFIC + NFIX_f2, ^b^Read count range (51, 148, 748, 444, respectively). *Ref.* reference

**Table 3:** Top 15 transcription factors enriched in T-47D cells

*TF name*	*Motif*	*Selection by:*	*TF binding sites*	*Ref. sites*	*Expected sites in ref.*	*Ratio of enrichment*	*Mean read count*
*MBD2*	MBD2_si	1, 2, 3, 4	101	6664	39.6	2.55	571
*TFAP2A*	AP2A_f2	1, 2, 3	115	10363	61.6	1.87	941
*E4F1*	E4F1_f1	2, 3	18	1750	10.4	1.73	310
*SP1*	SP1_f1	1, 2	392	41453	246.3	1.59	838
*CUX1*	CUX1_f1	1, 2, 3	13	1462	8.7	1.50	141
*E2F2*	E2F2_f1	1, 2	17	1941	11.5	1.47	215
*AHR*	AHR_si	1, 2, 3	9	1030	6.1	1.47	791
*SP2*	SP2_si	1, 2	140	16512	98.1	1.43	672
*CREB1*	CREB1_f1	1, 2, 3	23	2720	16.2	1.42	177
*CBFB*	PEBB_f1	1, 2, 3, 4	46	5461	32.4	1.42	457
*ZIC2*	ZIC2_f1	1, 2, 3	46	5487	32.6	1.41	118
*ZFX*	ZFX_f1	1, 2, 3	127	15650	93.0	1.37	287
*HIF1A*	HIF1A_si	1, 2, 3, 4	15	1890	11.2	1.34	1847
*E2F3*	E2F3_si	1, 2, 3	16	2019	12.0	1.33	322
*XBP1*	XBP1_f1	1, 3, 4	12	1545	9.2	1.31	22744

The ratio of enrichment is the result of dividing the number of TF binding sites by the number of expected sites. Motifs are derived from the HOCOMOCO database. *Ref.* reference

For more in-depth functional analysis we selected particular TFs from the top enriched candidates using the following criteria: 1) a binding motif with a quality category of A-C in the HOCOMOCO database, 2) relevance in the context of our model based on literature, 3) not a highly common regulator or part of a large TF family, and 4) high expression level of the TF (>1000 reads) in at least one cell line and differential expression between cell lines according to the RNA-seq. The last criteria was used to ensure methodological success in subsequent functional assays. With the criteria described above CBFB, HIF1A, and MBD2 were selected for further study. Of these, MBD2 had a large number of binding sites in the promoters of our DEGs while binding sites of the other two TFs were less widespread. In addition, SMAD4 was used as a positive control.

As SMAD4 is a known regulator of BMP signaling, we performed co-occurrence analysis of the binding sites between our three candidate TFs and the SMAD motifs. We found that the MBD2 motif was significantly co-localized with the GC-rich SMAD4 consensus motifs CGCC (*P* = 1.1e-9), GCCGnCGC (*P* = 1.3e-14), and GGCGCC (*P* = 2e-10). As binding sites for CBFB or HIF1A were less frequent across DEGs, statistical significance for co-localization with SMAD motifs could not be reliably evaluated. However, we did find several promoters where SMAD binding sites co-localized with these factors.

Silencing of selected TFs (SMAD4, CBFB, HIF1A, and MBD2) was then used to further evaluate their impact on BMP4 signaling. After 48 h of silencing, the cells were treated with BMP4 for 24 h and the mRNA levels of the validated DEGs were measured to assess whether the silencing influences BMP4 target gene expression (Fig. 4b and Additional file 8: Figure S3). Downregulation of SMAD4 was able to reverse the BMP4-mediated change in the expression of all the tested target genes in both MDA-MB-231 and T-47D cells (Fig. 4c) indicating that these expression changes are indeed transmitted via the canonical BMP pathway. For most of the target genes, MBD2 silencing led to abrogation of the BMP4-mediated induction in gene expression in both cell lines. In T-47D cells, similar data was also obtained for most of the genes upon CBFB (9/10) and HIF1A depletion (6/10). However in MDA-MB-231, silencing of HIF1A resulted exclusively in upregulation of the target genes and both enhanced and diminished expression was seen after CBFB downregulation. Of note, silencing of all of the TFs in T-47D cells led to the enhanced expression of the *DLL1* gene, which was consistent with it being downregulated upon BMP4 treatment. These data imply that the TFs may function as either repressors or enhancers of BMP4 target gene expression in a context-dependent manner.

## Discussion

We have previously characterized transcriptional responses of breast cancer cell lines to BMP4 by using microarray technology [14]. However, in that study we focused only on cells that respond to BMP4 by reduced proliferation. Efforts by others to examine BMP signaling target genes have concentrated exclusively on non-cancerous cells [18–20]. Here we set out to uncover the transcriptional responses of breast cancer cell lines with different phenotypes by using one cell line that responds to BMP4 by reduced proliferation (T-47D) and another that reacts with increased migration (MDA-MB-231). Being able to uncover the mechanisms of these two different responses is essential for the understanding of the role of BMP4 in breast cancer pathogenesis. To this end, we used a substantially new approach of combining DNase-seq, RNA-seq and functional experiments.

In order to find the early mediators of BMP4 response, we treated the cells with BMP4 or vehicle control for 3 h. At this time point, the canonical BMP pathway through SMAD1/5/9 is already activated [9]. The results of RNA-seq revealed that the cell lines responded to BMP4 by upregulating or downregulating a set of genes that were mostly cell line-specific, with only ten common DEGs identified. Consistent with the sequencing data, validation with qRT-PCR across multiple time points (3, 6, and 24 h) and five additional cell lines further confirmed in a wider context the existence of common BMP4 target genes as well as cell line-specific expression patterns. Of the ten shared DEGs, three were known BMP4 target genes (*ID1*-*3*) and two members of the BMP signaling pathway (*SMAD6*, *SMAD9*) [3]. The activation of the inhibitory *SMAD6* indicates a negative feedback loop, which in T-47D is reinforced by the upregulation of BMP antagonist *NOG* and the pseudoreceptor *BAMBI*. On the other hand, activation of the receptor-regulated *SMAD9* seems to point to a positive feedback loop, as alongside other R-SMADs, SMAD9 has been found to enhance BMP signaling [21, 22]. However, one study indicated that SMAD9 may have an inhibitory role in BMP signaling [23]. In any case, upregulation of *SMAD9* due to BMP4 treatment has also been recently reported in various cell types, for example in primary fibroblasts, hepatocellular carcinoma and melanoma cells [24].

To understand the function of the cell line-specific DEGs, we used GO analysis to segregate the DEGs into biological process categories and discovered that the results reflected the response of the cell lines to BMP4. Processes related to migration were enriched in the MDA-MB-231 cells, whereas more diverse responses were found in T-47D, including categories comprised of signaling, development and morphogenesis. These findings were corroborated by the DNase-seq data, where we found that BMP4-induced global open chromatin sites were enriched with the same biological categories that were found with RNA-seq data. While categories associated with signaling were observed in both cell lines, in MDA-MB-231 those related to migration were enriched. These data extend our previous results showing enrichment of differentially expressed genes in GO categories that were associated with the BMP4-induced decrease in proliferation [14]. Taken together, the different responses of the cell lines to BMP4 are reflected both at the transcriptional and chromatin levels.

In the analysis of TSS chromatin state we could observe changes in only a few of the genes that were differentially regulated by BMP4. This might be due to the fact that the 3 h stimulation of BMP4 is too short for most of the TSSs to change their chromatin status. Moreover, we could observe that in many cases the chromatin was already open at the TSS, in which case further changes are not needed to enhance the transcriptional activity. Together with the observation that there is a large variation between the chromatin status and gene expression when we extend the analysis to the whole set of protein-coding genes, it can be concluded that the chromatin state of TSSs explains the observed expression patterns only to a small extent. This result was not unexpected, as gene expression is also commonly regulated from regions located far from the TSS, such as enhancers [25, 26].

With genome-wide detection of open chromatin areas we noticed that BMP4 stimulation induces opening of the chromatin mostly in the intronic and intergenic regions. This is consistent with the fact that changes in the TSS and promoter regions were observed with only a few of the differentially expressed genes. Opening of the intronic sequences may indicate increased level of RNA polymerase activity at gene bodies. Chromatin opening at intergenic regions might suggest that additional regulatory control is being attained in large extent through distal regulatory elements such as enhancers and silencers. Thus, already at the early 3-h time point we are able to observe conformational changes that cells may utilize in more detailed regulation of the BMP response. Unfortunately, based on this analysis we were not able to define a specific transcription factor chromatin signature that could be used to define BMP-specific regulatory sequences. Hence detailed analysis of the putative enhancer regions would require more specific measurement data about the chromatin interactions in these cells.

To further characterize the regulation of BMP4 target genes, we analyzed transcription factor binding sites (TFBSs) in the open chromatin regions located on gene promoters. Among the top 15 enriched TFs, there were a few which had previously been linked to BMP target gene regulation. For example, XBP1 and RELA have been shown to be repressors of BMP target genes *Xvent-2* and *Id1*, respectively [27, 28]. Using enrichment of the TFBSs between cell lines as well as other criteria, we selected three TFs (CBFB, HIF1A, and MBD2) for functional characterization and silenced them in the two cell lines. In addition, we used SMAD4, a key component of the canonical BMP pathway, as our positive control and indeed SMAD4 was required for transcriptional regulation of all the BMP4 target genes in the assay. Although BMPs can signal through alternative pathways [7, 8], this result points to regulation through the canonical pathway. In contrast, the response to other transcription factors was more variable and cell line-specific.

MBD2 is a methyl-CpG-binding transcription factor that plays a role in development [29, 30]. Several studies have shown that MBD2 acts as a transcriptional repressor by recruiting co-repressor complexes to promoters, which in turn leads to formation of repressive chromatin through chromatin remodelling [31, 32]. However, there is also evidence that MBD2 can activate transcription by removing methylation from CpG islands located in promoters [33]. In both cell lines, MBD2 seemed to act mainly as an activator of transcription, although its role was more prominent in MDA-MB-231. In our analysis, MBD2 had a large number of binding sites across DEGs and it was highly expressed in both cell lines, consistent with the observed behavior in the silencing experiment. The key role of MBD2 in controlling the BMP4 response suggests that DNA methylation may be involved in BMP4 signaling.

The core-binding factor subunit beta (CBFB) together with the alpha subunit (RUNX1 or RUNX2) is involved in hematopoiesis and skeletal development [34, 35]. It was generally an activator of transcription in T-47D cells where its binding sites were enriched at DEGs promoters, but showed a less constant role in MDA-MB-231. Of note, CBFB has previously been found to influence BMP signaling in chondrocytes [36] and it has also been shown to have invasive properties in breast, prostate and ovarian cancer cells [37, 38].

Hypoxia-inducible factor 1-alpha (HIF1A) is a key regulator of the hypoxia response and has been linked to breast cancer progression [39]. In our binding site enrichment analysis, we observed that HIF1A binding sites are strongly depleted in MDA-MB-231 DEGs although HIF1A has a very high expression in this cell line. We also found that HIF1A was almost exclusively a transcriptional repressor of BMP4 target genes in MDA-MB-231 cells, whereas in T-47D cells it had either no effect or acted as an activator of transcription. Several hypoxia-related genes were found among DEGs, four in MDA-MB-231 cells (*BDKRB2*, *PDGFB*, *ANGPTL-4* and *UCN2*) and three in T-47D cells (*CBFA2T3*, *EGLN3* and *FLT1*). Interestingly, some of these genes have also been linked to cancer progression, for example HIF1A-dependent upregulation of *PDGFB* and *ANGPTL-4* promotes metastasis of hypoxic breast cancer cells [40, 41]. As an additional interesting aspect, HIF1A has been shown to activate BMP4 transcription in pulmonary arterial smooth muscle cells and in murine spleen and ES cells [42–44]. Together these findings support the view that HIF1A is indeed a cell type-specific repressor that controls a particular subset of BMP4-activated target genes.

## Conclusions

By combining genome-wide computational analyses and experimental data with functional validation, we were able to extend our knowledge about BMP4 signaling in breast cancer. This study demonstrates that the differential responses to BMP4, reduced proliferation and induced migration, seen in breast cancer cell lines in vitro, are reflected in the expression pattern of BMP4 target genes, thus allowing us to uncover regulatory mechanisms associated with these phenotypes.

By integration of chromatin state and transcription factor binding analyses with gene expression, we were able to identify candidate TFs involved in the regulation of BMP4 response. The function of these TFs was then tested by silencing experiments. From our three candidates, MBD2 emerged as a consistent activator of target gene expression in both cell lines, while HIF1A was shown to act as a repressor in cells with induced migration phenotype and CBFB as an activator, particularly in cells with reduced proliferation phenotype.

While understanding the full complexity of the regulation of BMP4 signaling will require more extensive data, analyses and experiments in wider contexts, our current study established the existence of phenotype-specific BMP response patterns in gene expression. Furthermore, we identified and experimentally validated cell type-specific downstream regulators of BMP signaling that relate to these expression patterns and thus to different in vitro phenotypes.

## Methods

### Breast cancer cell lines and treatments

Breast cancer cell lines BT-474, HCC-1954, MCF-7, MDA-MB-231, MDA-MB-361, MDA-MB-436, and T-47D as well as the normal immortalized mammary gland cell line MCF-10A were purchased from the American Type Culture Collection (ATCC, Manassas, VA, USA) and cultured according to the recommended conditions. The cell lines were authenticated by genotyping and were regularly tested for mycoplasma infection. Cells were seeded, allowed to adhere for 24 h, and treated with 100 ng/ml recombinant human BMP4 protein (R&D Systems, Minneapolis, MN, USA) or vehicle (BMP4 dilution solution). For RNA-seq and DNase-seq, one sample per cell line and treatment was used. Samples were collected 3 h after the treatment, based on our previous results showing SMAD1/5/9 protein phosphorylation [9] and gene expression changes by microarray analyses at this time point [14]. For qRT-PCR, samples representing three biological replicates were collected at indicated time points and pooled.

### RNA purification and sequencing library preparation

Total RNA was extracted from BMP4- and vehicle-treated cells using the Absolutely RNA miRNA kit (Agilent Technologies, Palo Alto, CA, USA) according to the manufacturer’s instructions. RNA quality was monitored using Agilent 2100 Bioanalyzer (Agilent Technologies). Sequencing libraries were generated using the TruSeq RNA Library Prep kit (Illumina Inc., San Diego, CA, USA) according to the manufacturer’s directions. Shortly, total RNA was enriched for Poly-A tails and then fragmented. Subsequently, RNA fragments were reverse transcribed into cDNA using random hexamer primers. Then, short fragments were purified and resolved with EB buffer for end reparation and adding poly(A). After that, the short fragments were ligated to sequencing adapters. Finally, suitable fragments were selected for the PCR amplification as templates and separated with agarose gel electrophoresis before sequencing.

### Preparation of DNase I-treated DNA and sequencing library

Cells were grown to 80–90% confluency, treated with BMP4 or vehicle for 3 h, and 3 × 10^7^ nuclei were isolated as previously described [16]. DNase I digestion was performed according to the protocol by Ling and Waxman [45]. First, 7.5 × 10^6^ nuclei were subjected to varying amounts of DNase I and different digestion times in order to optimize the conditions. The qPCR-based DNase hypersensitive site (DHS) cleavage assay [45] was performed using positive control primers surrounding known DHSs in the promoters of housekeeping genes and negative control primers from intergenic insensitive sites (Additional file 9: Table S8). Based on these analyses, 40 units of DNase I for 15 min was selected for the DNase I-treatment. The digestion reaction was followed by phenol-chloroform extraction and size fractionation of DNase I-released fragments by sucrose gradient ultracentrifugation. The DNA fraction with optimal enrichment of DHSs was chosen based on the qPCR-based fragment release assay [45]. Positive control primers were located inside known DHSs in the promoters of housekeeping genes and negative control primers in gene-free regions of different chromosomes (Additional file 9: Table S8). Fraction 7 gave optimal results (DNA fragments less than 1 Kb in size) in all cases and was therefore used for the subsequent steps. Libraries were generated using BGI’s in-house protocol. Shortly, 3′-dA overhangs were added and methylated sequencing adaptors were ligated to the DNA fragments. This was followed by PCR amplification and size selection to 200–400 bp, including the adaptor sequence. Undigested DNA from both cell lines was included as an input control.

### Deep sequencing

All library construction and deep sequencing steps were carried out at the Beijing Genomics Institute (BGI) (Hong Kong) according to their standard practice. Sequencing was performed on the Illumina HiSeq2000 platform (Illumina). Raw image files were processed by Illumina pipeline for base­calling with default parameters. Reads with too many N bases (>10%) or low base quality (>50% bases with base quality <5) were discarded. On average, we obtained 49 million 90 bp-long paired-end reads from the RNA-seq. For MDA-MB-231 cells, 49,403,872 reads were obtained for the BMP4-treated sample, while 49,424,070 reads resulted from the vehicle-treated sample. The equivalent read amounts for T-47D cells were 49,369,676 and 49,232,294, respectively. Sequencing of the DNase I-digested samples yielded on average 70 million 50 bp-long single-end reads. The specific read amount for MDA-MB-231 cells treated with BMP4 was 70,714,004, and 70,339,810 for vehicle-treated cells. The analogous numbers for T-47D cell line were 79,353,149 and 65,606,222.

### Read alignment and normalization of RNA-seq data

RNA-seq reads were aligned using TopHat2 against hg19 reference genome [46]. On average, we were able to align 96% of the reads. For MDA-MB-231 cells, 96.45% of the reads were aligned for the BMP4-treated sample, while 96.55% was the analogous value for vehicle-treated samples. The equivalent numbers for T-47D cell line were 96.33% and 96.02%. Raw expressions were calculated as simple read counts for composite genes constructed from the set of transcripts included in Gencode Genes version 19 [47] using the in-house tool Pypette (https://github.com/annalam/pypette) which is a toolkit built upon Samtools and Bedtools [48, 49]. Read counts were normalized across samples using median of ratios normalization implemented similarly as in DESeq2 R-package [50].

### Differential gene expression and GO analysis

In order to find differentially expressed genes (DEG) between BMP4- and vehicle-treated samples, log2 ratios were calculated. Genes having a log2 ratio absolute value of 0.75 or greater were considered differentially expressed. As additional criteria for DEGs, the absolute difference in read counts between the two treatments was required to be at least 50. Functional classification of the differentially expressed protein-coding genes was performed using the DAVID 6.8 version [51, 52].

### Survival analysis of DEGs

Each differentially expressed protein - coding gene was tested for association with the survival of breast cancer patients based on the gene expression data obtained from The Cancer Genome Atlas (TCGA) [53, 54]. The patients included in the dataset (*n* = 1212) were divided into low and high expression groups based on the median expression of the gene. The difference in the survival times between the two groups were tested using the logrank test and Benjamini-Hochberg correction was applied to the P-values. The survival analysis was implemented using the R-package RTCGA toolbox [55].

### Read alignment of DNase-seq data and detection of DNase hypersensitive sites (DHSs)

Reads were aligned using bowtie2 [56]. On average, we were able to align 97% of the reads. For MDA-MB-231 cells, 97.49% of the reads were aligned for the BMP4-treated sample, while 97.40% was the analogous value for vehicle-treated samples. The equivalent numbers for T-47D cell line were 97.45% and 97.75%. DNase hypersensitive sites (DHSs) were detected using DFilter [57]. The standard deviation was set to 2, bin size 100 bp and kernel size 50. In addition, the refine parameter was used. To mitigate the effects of mappability and coverage bias samples that had not been treated with DNase I were used as input controls. To remove likely false positives, all DHSs that were covered by less than 20 reads in sample or in input control were omitted from further analysis. Similarly, DHSs located in positions overlapping blacklisted regions collected by the ENCODE consortium were filtered out [58]. Additionally, adjacent DHSs (distance between peaks 100 bp or less) were merged together. The merged DHSs were annotated by their association to genomic features obtained from Gencode Genes version 19 using Bedtools [49].

### Benchmarking the detected DHSs against available ENCODE datasets

To confirm the consistency of the results of our DHS detection in comparison to available ENCODE data, two DNase-seq datasets, each consisting of two T-47D untreated replicates, were retrieved from GEO (accession numbers: GSM816673 and GSM1024762) [59, 60]. The alignment of reads and peak detection were done according to the workflow described above. Further on, we refer to these datasets by their ENCODE biosample identifiers: ENCSR000ELT and ENCSR000EQB.

### Finding differential DHSs (∆DHS) and functional enrichment analysis

In order to describe the change in chromatin hypersensitivity, DHS change scores (∆DHS) were calculated between the two conditions using a slightly modified formula to the one introduced by He et al. [61]. The DHS change score for i:th DHS was calculated using the following formula:

$$ \varDelta DHS=\sqrt{n_i^{treated}/\left({\displaystyle \sum_{k=1}^m{n}_k^{treated}}\right)}/m-\sqrt{n_i^{vehicle}/\left({\displaystyle \sum_{k=1}^m{n}_k^{vehicle}}\right)/m} $$

,where n_i_^treated^ is the number of reads mapped to DHS in the treated sample and n_i_^vehicle^ is the number of reads mapped to the DHS in the vehicle sample.

The DHSs having ∆DHS equal or greater than 0.20 were selected for enrichment analysis. The analysis was conducted with GREAT version 3.0.0 [17] using the default parameters. The results were ranked and selected based on the binomial test such that all FDR adjusted p-values were required to be less than 0.05. To filter out overly generic ontology terms all categories including more than 1000 genes were filtered out from the final results of the analysis. In addition, too small categories including less than ten genes were removed.

### Calculation of DNase coverage of TSS and correlation with gene expression

All possible transcription start sites (TSS), collected from GENCODE transcripts corresponding to protein - coding genes, were extended 1000 bases to both directions. The coverage was calculated for each of these extended TSS regions, which we further refer to simply as TSS. Furthermore, to obtain a single coverage value to describe the openness of the TSS for each protein - coding gene, a weighted sum of the coverages of the TSSs over all the transcripts associated to that gene was calculated. The weight for each transcript’s TSS was determined based on the ratio of the estimated expression of the transcript and the total expression of the gene, which was calculated using RSEM [62]. In case the gene was not expressed in either vehicle or stimulated condition, the same ratio which was observed in the other condition was used. Moreover, if the gene was not expressed in either condition, the maximum TSS coverage over all the transcript’s TSSs was used as the representative coverage of the TSS of the gene. For visualization purposes, the chromatin status of each gene’s TSS was classified into two categories: closed or open. A TSS was considered to be closed if its coverage belonged to the 1. quintile of the TSS coverages of all genes, in that particular cell line and condition. Otherwise the TSS was considered to be open. Each TSS was associated to the corresponding normalized expression value of the gene, which had been obtained by dividing the expression value obtained after median of ratios normalization by the gene’s total exon length.

### Prediction of transcription factor binding sites in promoters of upregulated genes

In order to find potential transcriptional regulators of BMP4 response, DHSs overlapping proximal promoters (2000 bp upstream regions) of upregulated genes in MDA-MB-231 and T-47D cell lines were scanned with Position Weight Matrices (PWMs). Due to the low signal-to-noise ratio observed in T-47D samples some DHS regions might be narrower or even absent in the data as can be concluded by comparing the promoter-associated DHS regions between our T-47D samples to untreated ENCODE DNase-seq datasets described earlier (see Additional file 5: Table S5). In order to increase the robustness of our analysis we created a composite dataset by taking the union of all promoter-associated DHSs across our samples and all untreated ENCODE samples. The PWMs were created from the curated collection of Weighted Position Count Matrices (WPCMs) retrieved from HOCOMOCO database (version 9) [63]. The PWMs were calculated from weighted matrices of positional counts (WPCM) using the following formula previously introduced by Makeev et al. [64]:

$$ {S}_{b,i}= ln\frac{x_{b,i}+a{q}_b}{\left(W+a\right){q}_b} $$

,where **x**_**b,i**_ is the positional count of base **b** in the i:th column of WPCM, **W** is the total weight of the WPCM, **a** is the pseudo count defined as **ln(W)** and q_b_ is the background frequency of base **b** calculated across all the analyzed sequences.

The score for transcription factor binding match **(M**_**j**_ **)** was obtained for each position within the peaks by scanning the sequence using the previously defined PWMs. The score for position **j** when scanning with PWM **S** of length **w** is calculated as follows:

$$ {M}_j={\displaystyle \sum_{i=0}^{w-1}{S}_{b\left(i+j\right)},i} $$

We considered a PWM to be a match if the PWM score had a *p*-value less or equal than 0.001. The score thresholds corresponding to the used p-value cut-off were determined using MACRO-APE [65].

### Finding enriched and depleted transcription factor binding sites (TFBS) in promoters of upregulated genes

In order to find enriched transcription factor binding sites a background model was generated by selecting the DHSs of all proximal promoters not included in the set of promoters of upregulated genes as the background set. The background set was scanned for transcription factor binding sites as above. Based on the background set, the expected number of transcription factor sites were calculated for the promoter sets of upregulated genes for MDA-MB-231 and T-47D by first dividing the total number of found TFBSs by the cumulative length of the scanned DHSs in the background set and then multiplying this ratio by the cumulative length of the scanned DHSs in the corresponding promoter set of upregulated genes. The ratio of enrichment was then calculated by dividing the observed TFBSs by the number of expected TFBSs.

### Co-localization enrichment analysis of selected TFs and known consensus SMAD4-motifs

Six elements including: CAGACA, GTCT, CAGC, CGCC, GGCGCC and GCCGnCGC which have been previously reported as Smad-binding elements (SBEs) [66–69] were selected for co-localization enrichment analysis. The analysis was conducted such that all TFBSs which fall within 200 bp distance of a consensus motif were considered as co-localized with the motif. The binomial test was used to test for enrichment.

### qRT-PCR

Quantitative real-time PCR was performed using the Lightcycler 2.0 instrument (Roche, Mannheim, Germany) with LightCycler® TaqMan® Master reaction mix (Roche). Universal probe library (UPL) probes (Roche) and associated primers (Sigma-Aldrich, St. Luis, MO, USA) were used for most of the genes, and the LightCycler FastStart DNA Master SYBR Green I assay (Roche) for the rest. Roche’s Reference Gene Assay for HPRT was used for normalization. Primer sequences and probe information are given in Additional file 9: Table S9.

### Transcription factor silencing

Transfections to silence the selected TFs in MDA-MB-231 and T-47D cells were performed on 24-well plates using 10 nM siRNA (siGENOME SMARTpool siRNAs, Dharmacon, Lafayette, CO, USA) and either the Interferin reagent (Polyplus-Transfection, SanMarcos, CA, USA) or DharmaFECT (Dharmacon) according to manufacturer’s instructions. An ON-TARGETplus Non-targeting Control Pool was used as control (Dharmacon). The knock-down of TFs was confirmed by qRT-PCR and at least 80% reduction in mRNA level was considered as adequate silencing. Forty-eight hours after the transfection, the cells were treated with 100 ng/ml BMP4 or vehicle for 24 h. Cell samples were collected by pooling three identically treated wells and RNA was isolated for subsequent qRT-PCR analyses.

## Additional files

Additional file 1: Figure S1. Relationship between chromatin status of TSS and gene expression. The boxplots illustrate the distribution of DNase-seq read coverage at TSS for protein-coding genes at five different levels of gene expression, which were determined by division of expressions into quintiles. Panel A shows the results obtained from untreated MDA-MB-231 cells and panel B the corresponding results for untreated T-47D cells. Panels C and D illustrate the difference between non-expressed and differentially expressed (protein - coding) genes in terms of the chromatin status at TSS in vehicle-treated samples of MDA-MB-231 and T-47D cells, respectively. In both cell lines, chromatin is clearly open at the TSS of differentially expressed genes before the stimulation with BMP4. (PDF 2463 kb)
Additional file 2: Table S1. Differentially expressed genes after BMP4 treatment in MDA-MB-231 cell line. Ensembl IDs, read counts, fold changes and Log2 ratios are shown. The genes are arranged in order from the largest to smallest Log2 ratio, first upregulated genes and then downregulated genes. (XLSX 20 kb)
Additional file 3: Table S2. Differentially expressed genes after BMP4 treatment in T-47D cell line. Ensembl IDs, read counts, fold changes and Log2 ratios are shown. The genes are arranged in order from the largest to smallest Log2 ratio, first upregulated genes and then downregulated genes. (XLSX 34 kb)
Additional file 4: Table S3. Survival analysis of DEGs in MDA-MB-231. **Table S4**. Survival analysis of DEGs in T-47D. Each differentially expressed protein - coding gene was tested for possible association with the survival of breast cancer patients based on the gene expression data obtained from The Cancer Genome Atlas (TCGA). Blue background indicates DEGs shared by both cell lines. Benjamini-Hochberg corrected P-values are shown. No diff. = no association with survival. Not available = not found in TCGA data. (XLSX 16 kb)
Additional file 5: Table S5. Comparison between our T-47D DNase-seq data and analogous data from ENCODE. DNase-seq peaks from promoter regions in BMP4-treated and vehicle-treated T-47D cells were compared to DNase-seq data of T-47D promoters from ENCODE (ENCSR000ELT replicates 1 and 2; ENCSR000EQB replicates 1 and 2). The table shows the percentage of shared peaks between pairs of samples. A high percentage of the peaks identified in our data are also present in ENCODE samples (cells B6-E7). (XLSX 8 kb)
Additional file 6: Figure S2. Shared DNase-seq peaks between BMP4 and vehicle samples. The number of shared peaks and the number of unique peaks in each treatment group are indicated in the Venn diagram. (PDF 153 kb)
Additional file 7: Table S6. MDA-MB-231 GREAT analysis. **Table S7**. T-47D GREAT analysis. Enrichment of open chromatin peaks using GREAT. Red color denotes categories that are the same as in the RNA-seq ontology analysis. (XLSX 146 kb)
Additional file 8: Figure S3. Transcription factor validation. The chosen TFs were silenced and then treated with BMP4 before measuring target gene expression using qRT-PCR. The transcription factor and cell line in question is stated at the beginning of each page. DLL, which is downregulated in T-47D upon BMP4 treatment, is circled with red. (PDF 256 kb)
Additional file 9: Table S8. Primers used for DNase-seq. **Table S9**. Primer sequences for qRT-PCR based expression analyses of BMP4 target genes and transcription factors. (DOCX 19 kb)

## Abbreviations

BMP4: Bone morphogenetic protein 4
DEG: Differentially expressed gene
DHS: DNase hypersensitive site
GO: Gene ontology
PWM: Position weight matrices
TF: Transcription factor
TFBS: Transcription factor binding site
TSS: Transcription start site
WPCM: Weighted position count matrices
