# Supplementary figures and images for: Integrated RNA-seq and DNase-seq analyses identify phenotype-specific BMP4 signaling in breast cancer
Source: BMC Genomics. 2017 Jan 11;18:68. doi: 10.1186/s12864-016-3428-1 (PMC5225521; doi:10.1186/s12864-016-3428-1)

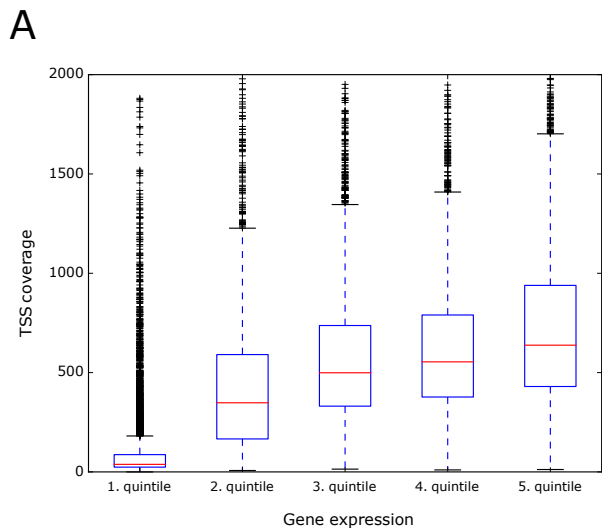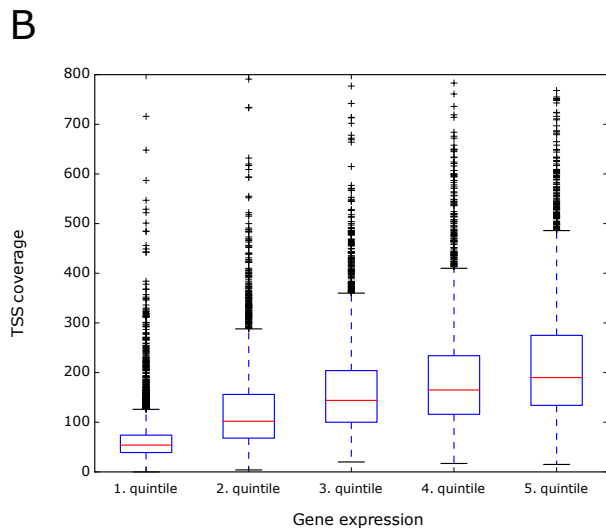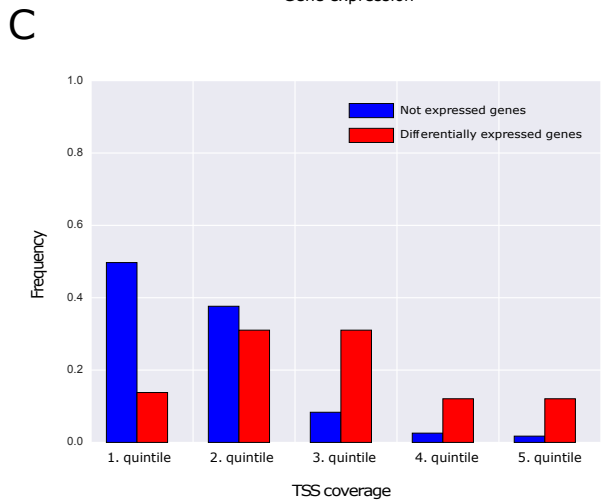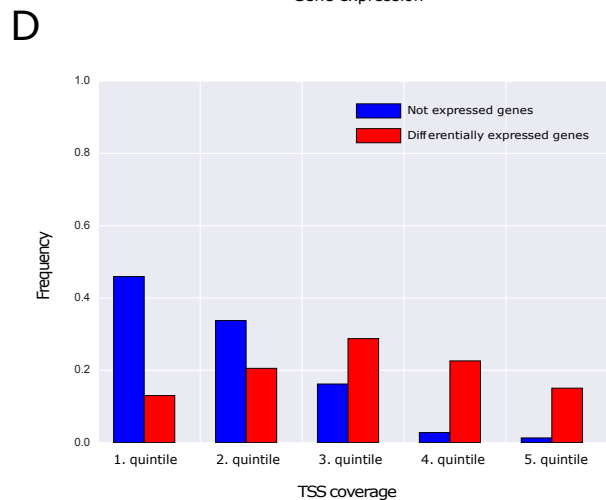

Supplement: Additional file 1: Figure S1. — Relationship between chromatin status of TSS and gene expression. The boxplots illustrate the distribution of DNase-seq read coverage at TSS for protein-coding genes at five different levels of gene expression, which were determined by division of expressions into quintiles. Panel A shows the results obtained from untreated MDA-MB-231 cells and panel B the corresponding results for untreated T-47D cells. Panels C and D illustrate the difference between non-expressed and differentially expressed (protein - coding) genes in terms of the chromatin status at TSS in vehicle-treated samples of MDA-MB-231 and T-47D cells, respectively. In both cell lines, chromatin is clearly open at the TSS of differentially expressed genes before the stimulation with BMP4. (PDF 2463 kb) [file 12864_2016_3428_MOESM1_ESM.pdf]

MDA-MB-231 peak distribution

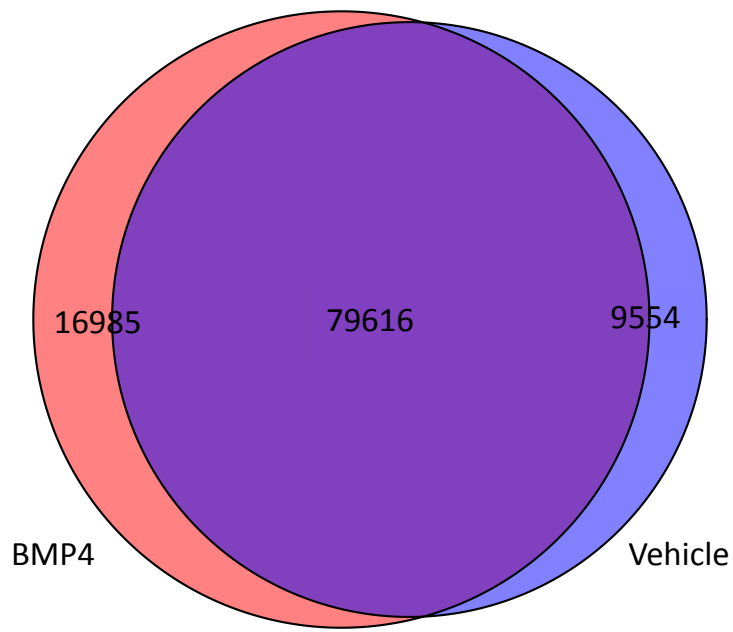

T-47D peak distribution

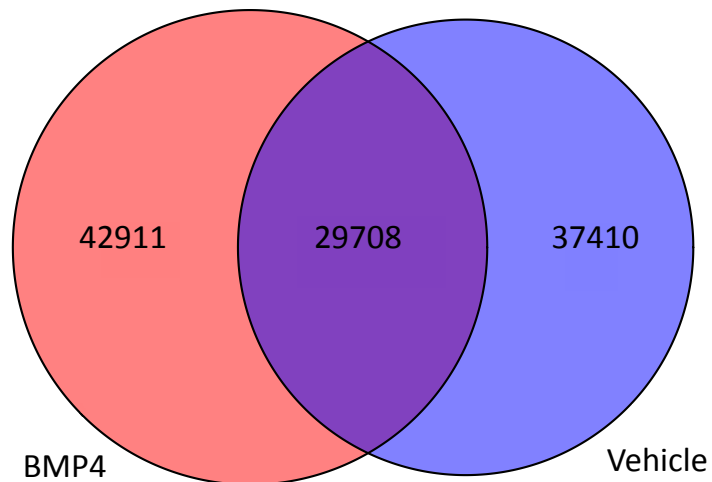

Supplement: Additional file 6: Figure S2. — Shared DNase-seq peaks between BMP4 and vehicle samples. The number of shared peaks and the number of unique peaks in each treatment group are indicated in the Venn diagram. (PDF 153 kb) [file 12864_2016_3428_MOESM6_ESM.pdf]
