# Supplementary material for: Integrated RNA-seq and DNase-seq analyses identify phenotype-specific BMP4 signaling in breast cancer
Source: BMC Genomics. 2017 Jan 11;18:68. doi: 10.1186/s12864-016-3428-1 (PMC5225521; doi:10.1186/s12864-016-3428-1)
Supplement: Additional file 8: Figure S3. — Transcription factor validation. The chosen TFs were silenced and then treated with BMP4 before measuring target gene expression using qRT-PCR. The transcription factor and cell line in question is stated at the beginning of each page. DLL, which is downregulated in T-47D upon BMP4 treatment, is circled with red. (PDF 256 kb) [file 12864_2016_3428_MOESM8_ESM.pdf]

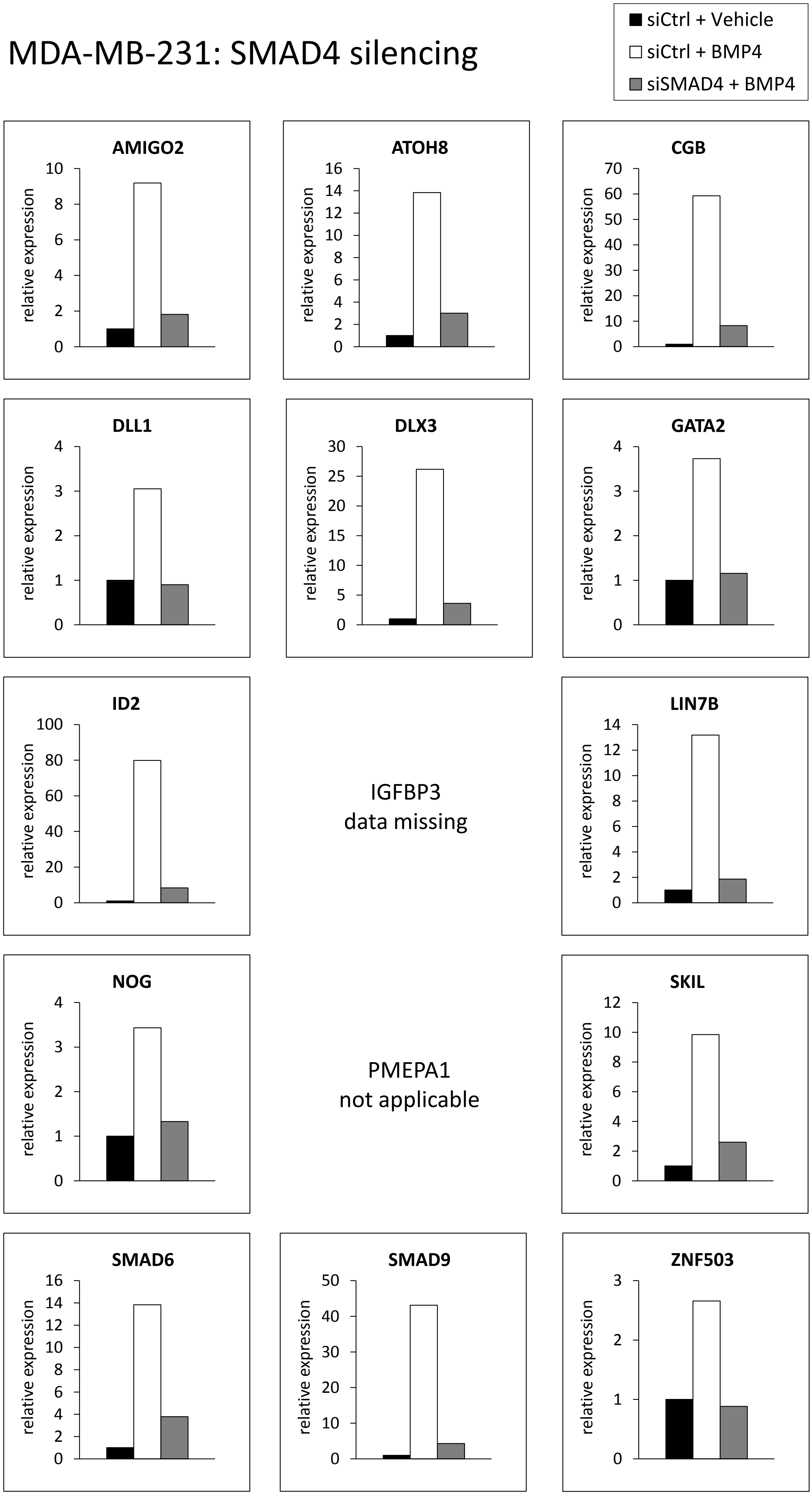
MDA-MB-231: SMAD4 silencing
siCtrl + Vehicle
siCtrl + BMP4
siSMAD4 + BMP4
AMIGO2
ATOH8
CGB
DLL1
DLX3
GATA2
ID2
IGFBP3
data missing
LIN7B
NOG
PMEPA1
not applicable
SKIL
SMAD6
SMAD9
ZNF503
relative expression

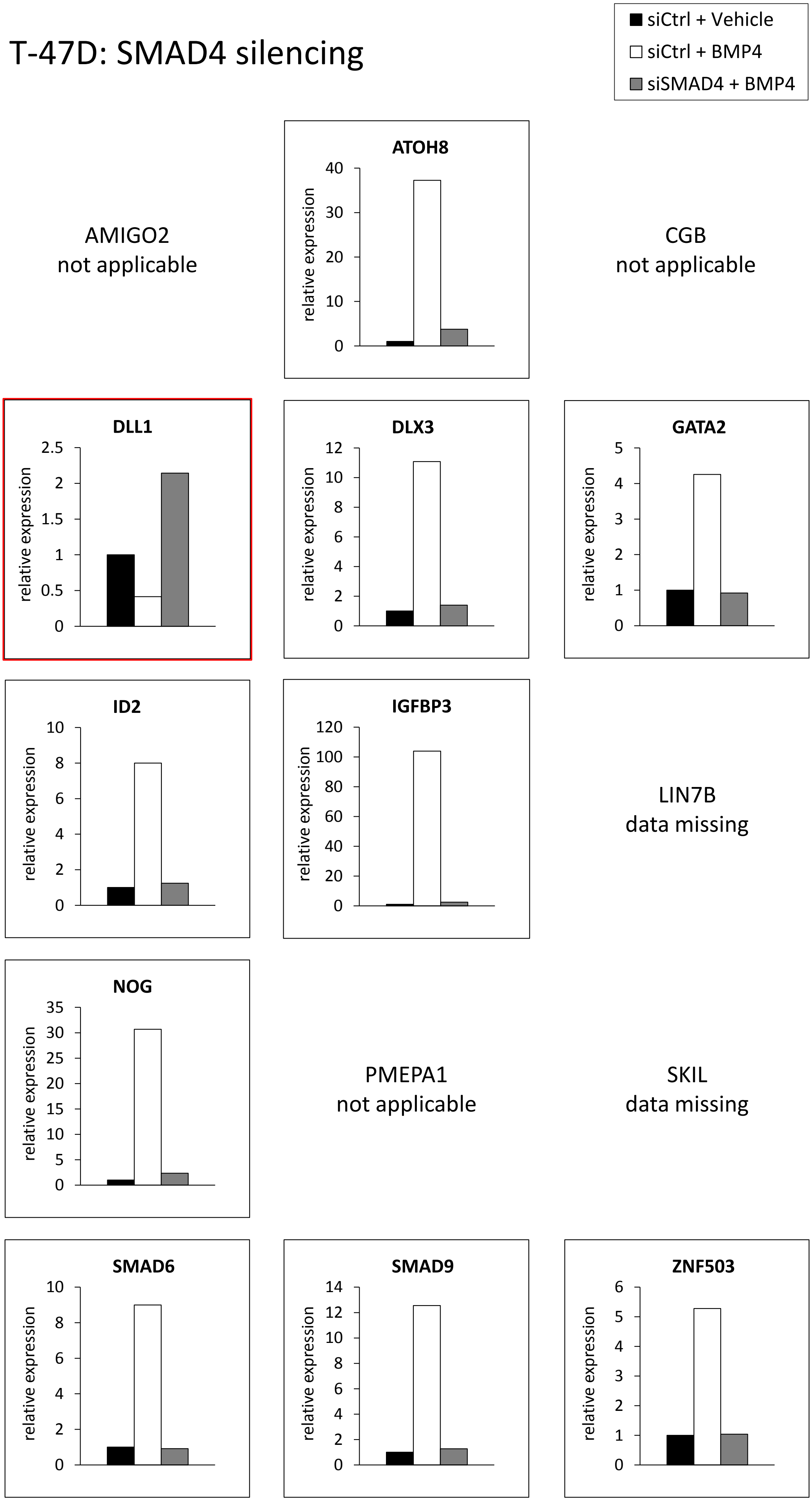
T-47D: SMAD4 silencing
siCtrl + Vehicle
siCtrl + BMP4
siSMAD4 + BMP4
AMIGO2
not applicable
ATOH8
CGB
not applicable
DLL1
DLX3
GATA2
ID2
IGFBP3
LIN7B
data missing
NOG
PMEPA1
not applicable
SKIL
data missing
SMAD6
SMAD9
ZNF503
relative expression

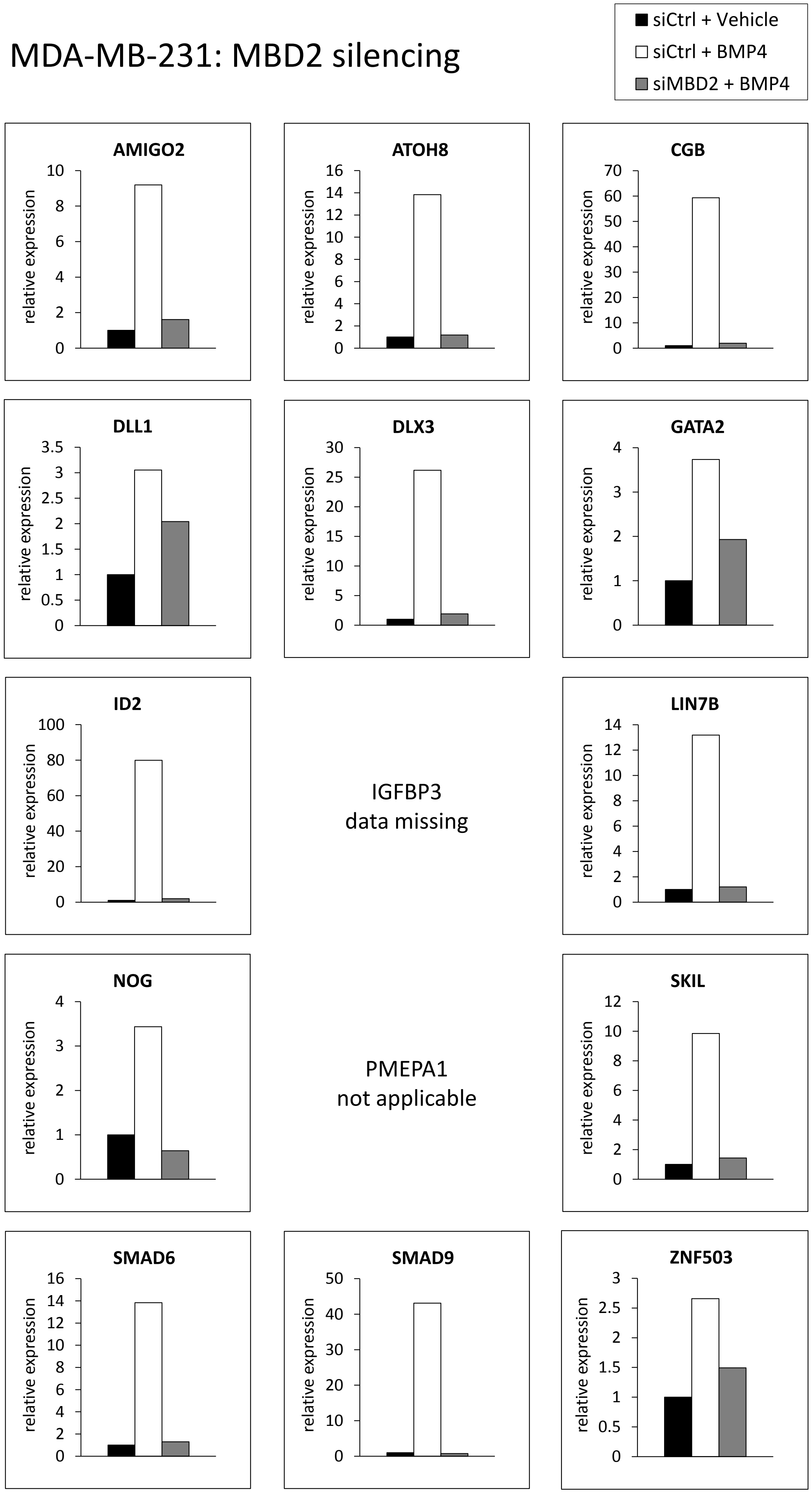
MDA-MB-231: MBD2 silencing
siCtrl + Vehicle
siCtrl + BMP4
siMBD2 + BMP4
AMIGO2
ATOH8
CGB
DLL1
DLX3
GATA2
ID2
IGFBP3
data missing
LIN7B
NOG
PMEPA1
not applicable
SKIL
SMAD6
SMAD9
ZNF503
relative expression

T-47D: MBD2 silencing

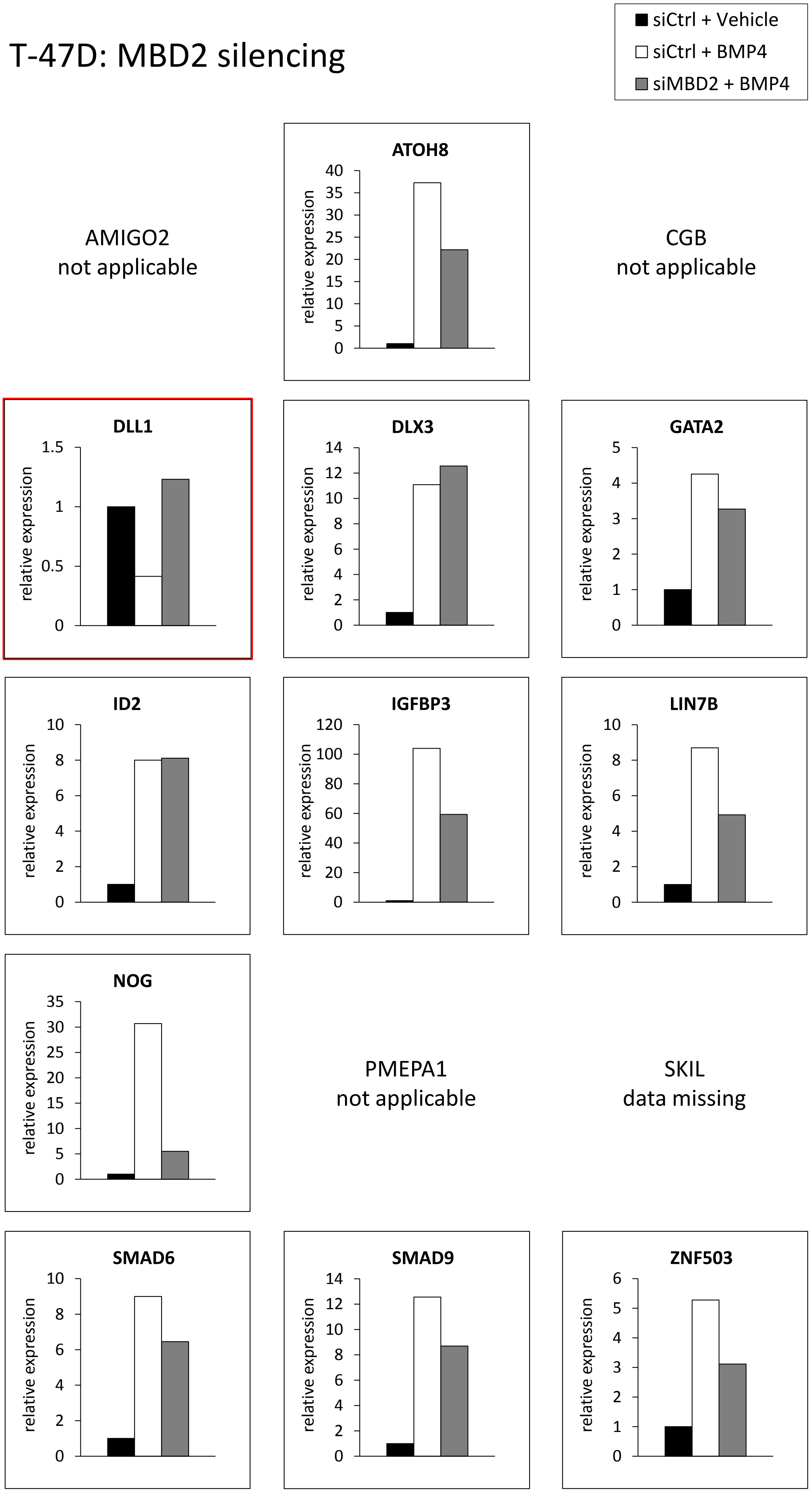

MDA-MB-231: CBFB silencing

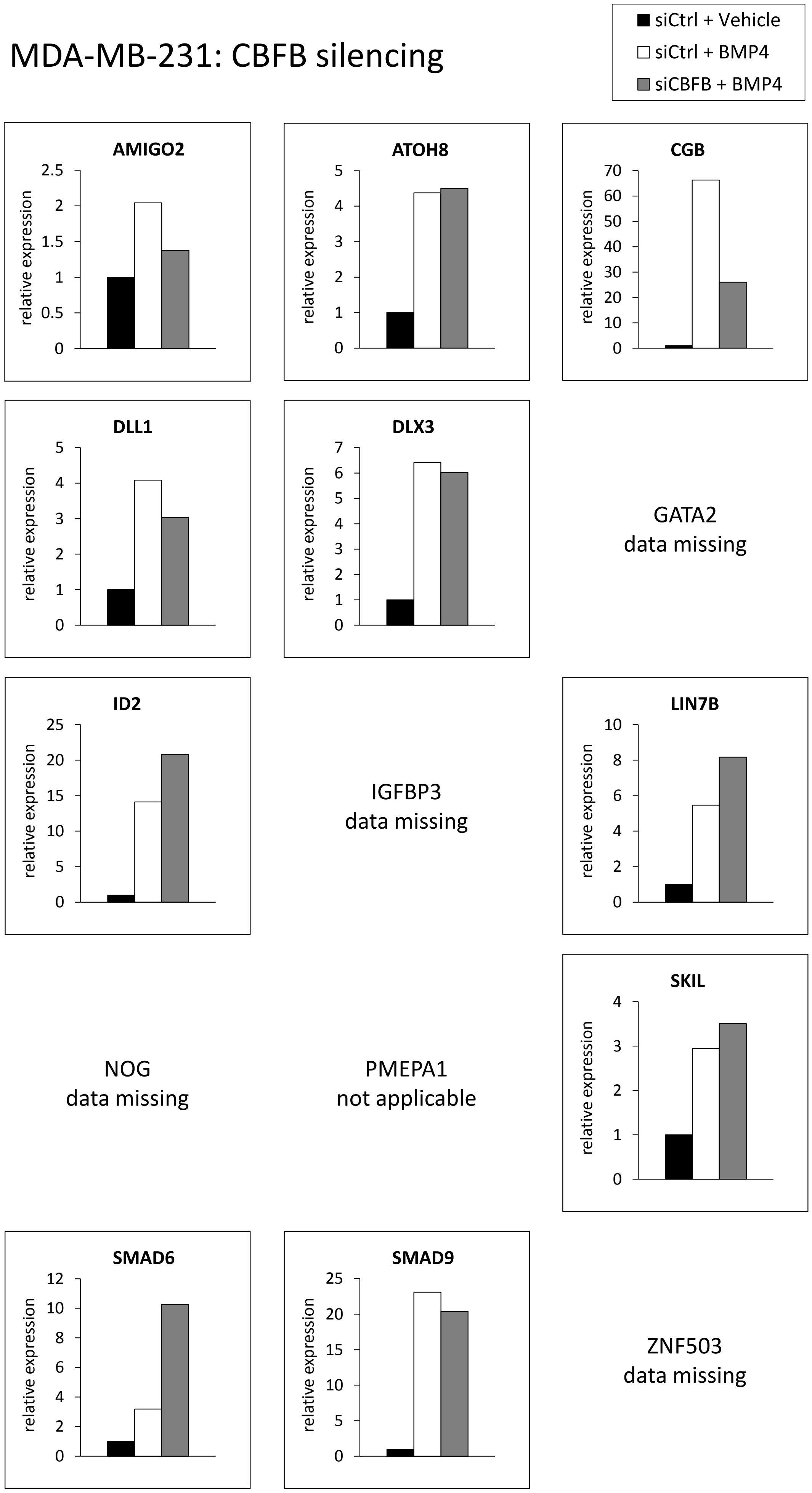

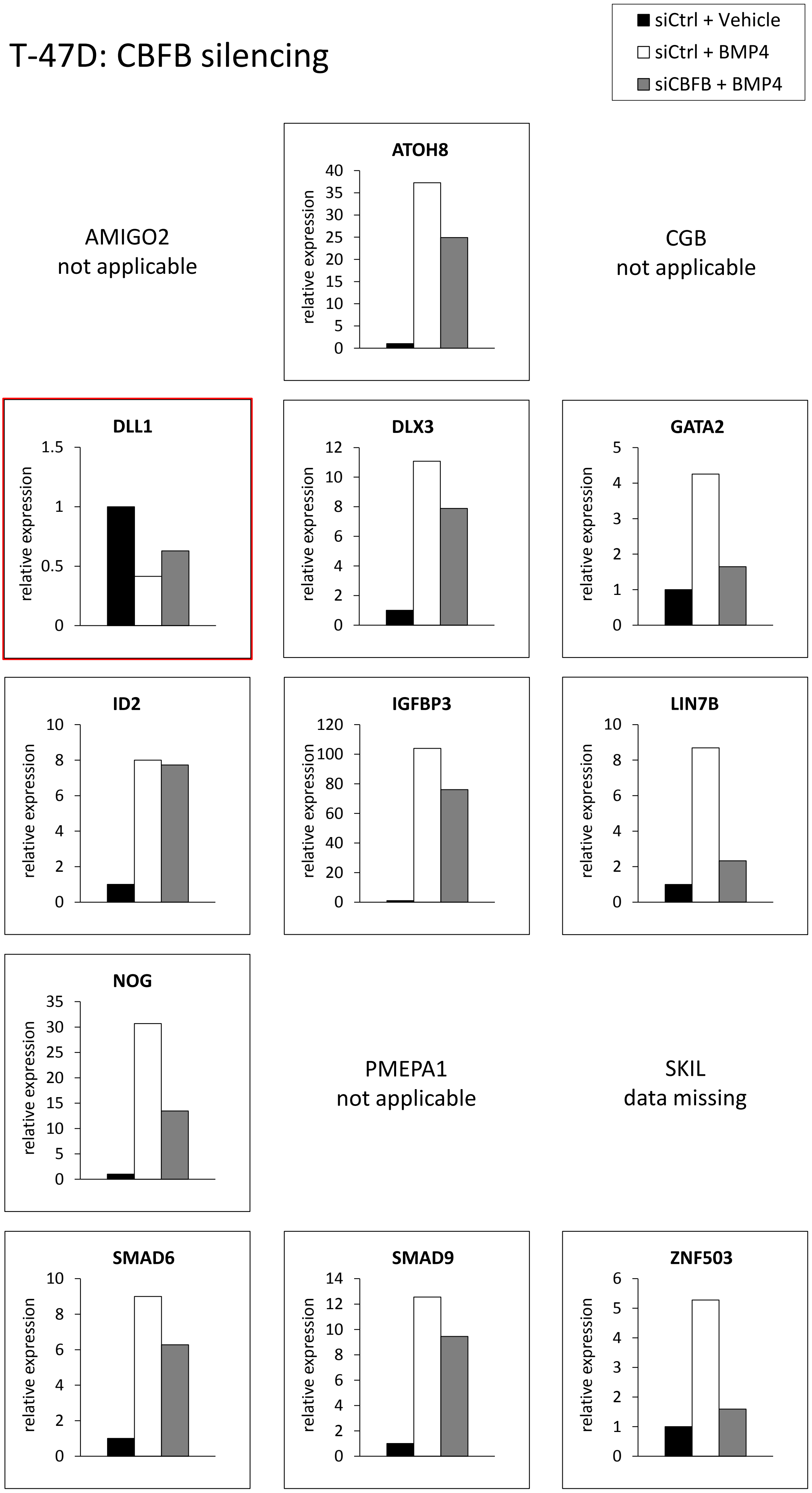
T-47D: CBFB silencing
siCtrl + Vehicle
siCtrl + BMP4
siCBFB + BMP4
AMIGO2
not applicable
ATOH8
relative expression
0 5 10 15 20 25 30 35 40
CGB
not applicable
DLL1
relative expression
0 0.5 1 1.5
DLX3
relative expression
0 2 4 6 8 10 12
GATA2
relative expression
0 1 2 3 4 5
ID2
relative expression
0 2 4 6 8 10
IGFBP3
relative expression
0 20 40 60 80 100 120
LIN7B
relative expression
0 2 4 6 8 10
NOG
relative expression
0 5 10 15 20 25 30 35
PMEPA1
not applicable
SKIL
data missing
SMAD6
relative expression
0 2 4 6 8 10
SMAD9
relative expression
0 2 4 6 8 10 12 14
ZNF503
relative expression
0 1 2 3 4 5 6

MDA-MB-231: HIF1A silencing

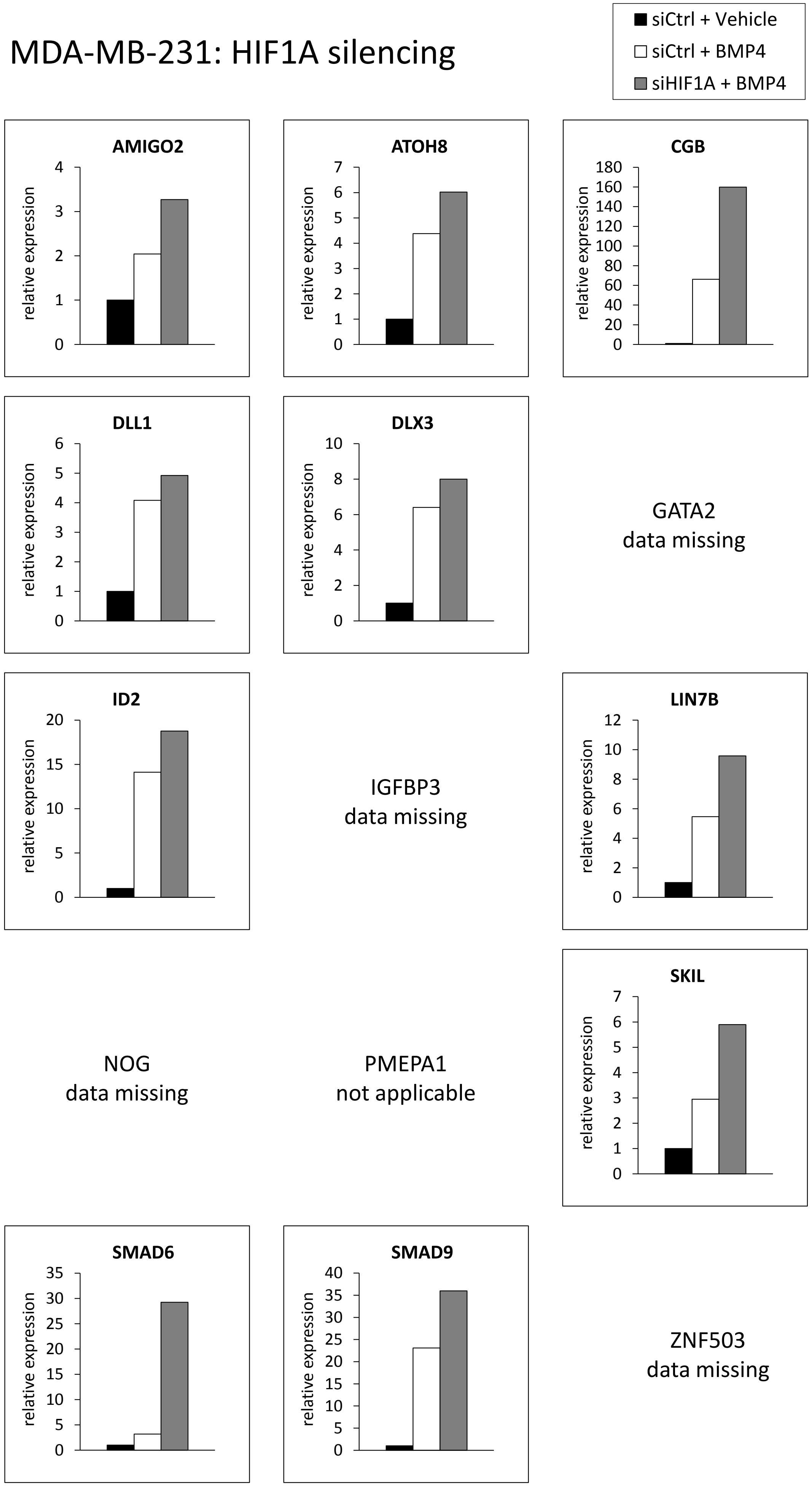

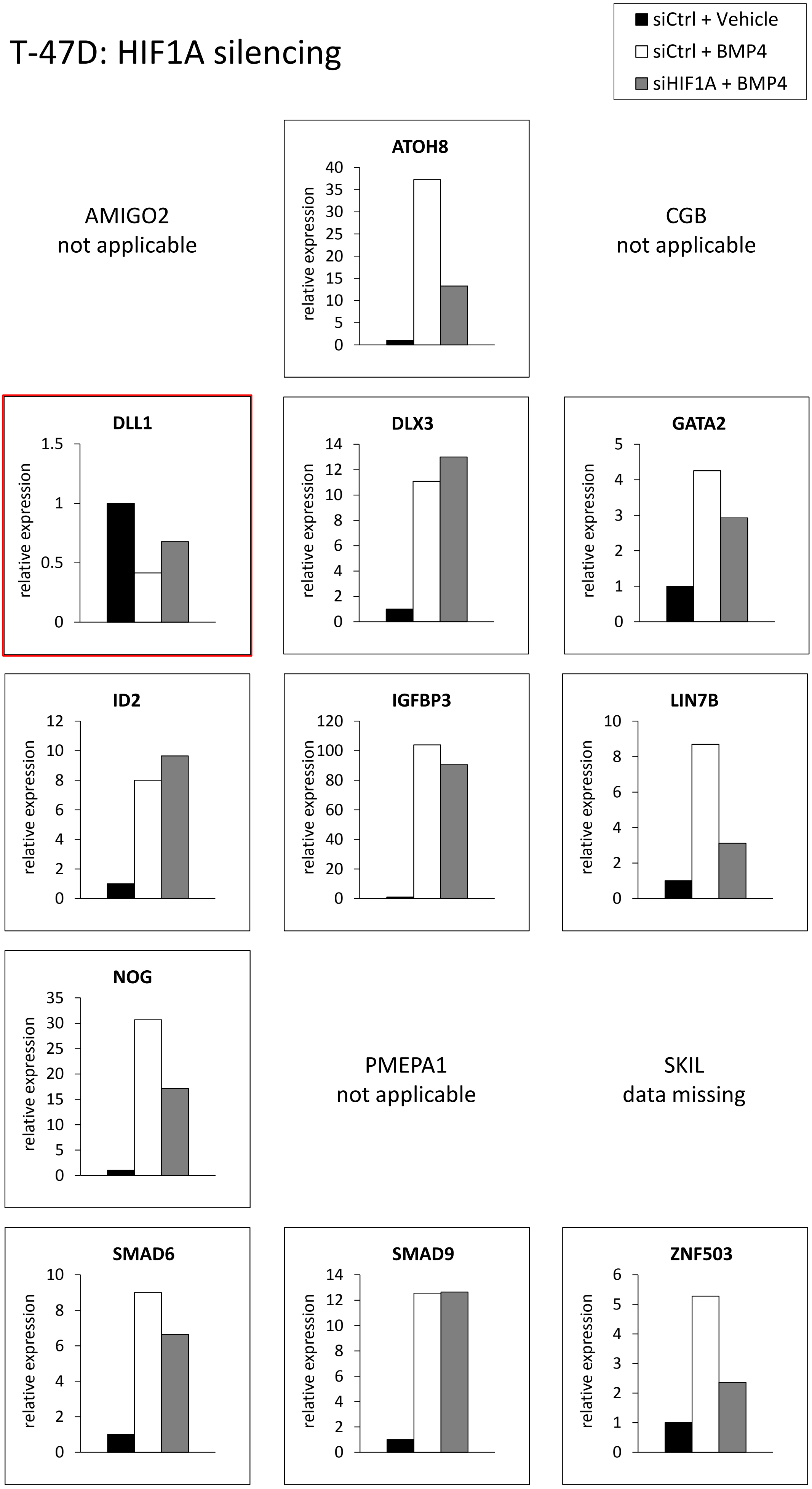
T-47D: HIF1A silencing
siCtrl + Vehicle
siCtrl + BMP4
siHIF1A + BMP4
AMIGO2
not applicable
ATOH8
CGB
not applicable
DLL1
DLX3
GATA2
ID2
IGFBP3
LIN7B
NOG
PMEPA1
not applicable
SKIL
data missing
SMAD6
SMAD9
ZNF503
relative expression
