# Supplementary material for: Integrated RNA-seq and DNase-seq analyses identify phenotype-specific BMP4 signaling in breast cancer
Source: BMC Genomics. 2017 Jan 11;18:68. doi: 10.1186/s12864-016-3428-1 (PMC5225521; doi:10.1186/s12864-016-3428-1)
Supplement: Additional file 9: Table S8. — Primers used for DNase-seq. Table S9. Primer sequences for qRT-PCR based expression analyses of BMP4 target genes and transcription factors. (DOCX 19 kb) [file 12864_2016_3428_MOESM9_ESM.docx]

Table S8. Primer sequences used in sample preparation for DNase-seq qPCR. All assays were done with SYBR Green.

| *Primer pair* | *Forward primer (5'-3')* | *Reverse primer (5'-3')* | *Amplicon* |
| --- | --- | --- | --- |
| *Primer sequences for DHS cleavage assay* | | | |
| LGUSB | CGTCGGTTGTCAGAGAAGT | CCTGCAACACCAAGAGGGA | 696 |
| LB2M | CAGAAGTTCTCCTTCTGCTAGGT | TGGAGAAGGGAAGTCACGGA | 644 |
| LNegC9 | AACCCCAAGGCATCCAAACA | TTCTCTGCCTGCCAAAGTCC | 682 |
| LNegC20 | TTGCCTTGTTCCCAGCAGAA | GCCACATAGCCTTCCAACCT | 672 |
| *Primer sequences for fragment release assay* | | | |
| sB2Mprom | CTGGCTTGGAGACAGGTGA | CCCAGCCAATCAGGACAAGG | 52 |
| sGUSBprom | CCAGAACAGAACCCCTGAGA | CTCCTTGAAGAAACAGGGGGAT | 50 |
| sPPIAprom | TCCGTCTATAGGCCAGATGC | CCAATCGGGTCTGCGACTT | 54 |
| sNegC2 | GCCAGTTCATGCTGTCTACCA | CGCAGTTCAGCAAAGGGAAG | 53 |
| sNegC9 | AGTGTGTTCAGAGTTGGAAGGA | AGACTGGAAGACAGGGAGAA | 53 |
| sNegC20 | AACAGGTGGAAAGAGCCACA | TCACTCCACTGTTGTCCACT | 50 |

Table S9. Primer sequences for BMP4 target genes and transcription factors. qPCR assays were performed either with Roche UPL probe system (probe number provided) or SYBR Green.

| *Primer pair* | *Forward primer (5'-3')* | *Reverse primer (5'-3')* | *Probe* |
| --- | --- | --- | --- |
| *Primer sequences for BMP4 target genes* | | | |
| AMIGO2 | CCCCAGCACCGTTCAC | ACGACATTATGGTCGCCTCTG | - |
| ATOH8 | GAGGGACGTGCCAAGAAG | TCAGCGAGCTCACCTTGTC | 12 |
| CGB | CTACTGCCCCACCATGACCC | GGTAGTTGCACACCACCTGA | - |
| DLL1 | CTTCCCCTTCGGCTTCAC | GGGTTTTCTGTTGCGAGGT | 2 |
| DLX3 | GAGCCTCCTACCGGCAATAC | TCCTCCTTCACCGACACTG | 26 |
| GATA2 | CTACAGCAGCGGACTCTTCC | ACTCCCGGCCTTCTGAACA | - |
| ID2 | ATATCAGCATCCTGTCCTTGC | AAAGAAATCATGAACACCGCTTA | 5 |
| IGFBP3 | GGATAAGTATGGGCAGCCTCT | TGAGCTCCACATTAACCTTGC | 12 |
| LIN7B | GCTTTATGACACGCTGGACA | GCTCCACTACCCTGGGATG | 8 |
| NOG | TAGAGTTCTCCGAGGGCTTG | CTCCGCAGCTTCTTGCTTAG | 37 |
| PMEPA1 | GCACAGTGTCAGGCAACGG | AGATGGTGGGTGGCAGGTC | - |
| SKIL | GAGGCTGAATATGCAGGACAG | CTTGCCTATCGGCCTCAG | 13 |
| SMAD6 | GGGCCCGAATCTCCGC | AGAATTCACCCGGAGCAGTG | - |
| SMAD9 | GCATTAACCCTTACCACTACCG | GAGCTGGGGGTTATATTCACTG | 16 |
| ZNF503 | ATTTTGCACCCCGAGTACCT | CTTCCCGATCTGCGAACA | 8 |
| *TF primer sequences* | | | |
| CBFB | ATGGTATGGGCTGTCTGGAG | TCAAAGGCCTGTTGTGCTAA | 88 |
| HIF1A | GATAGCAAGACTTTCCTCAGTCG | TGGCTCATATCCCATCAATTC | 64 |
| MBD2 | ACGAATGAATGAACAGCCACG | TGGACCAACTCCTTGAAGACC | - |
| SMAD4 | TGTGTTACCATACAGAGAACATT | GGGCATAGATCACATGAGGAA | 83 |
